# Impact of public smoking bans on children’s exposure to tobacco smoke at home: a systematic review and meta-analysis

**DOI:** 10.1186/s12889-018-5679-z

**Published:** 2018-06-21

**Authors:** Sarah Nanninga, Stefan K. Lhachimi, Gabriele Bolte

**Affiliations:** 1 Institute of Public Health and Nursing Research, Department of Social Epidemiology, University of Bremen, Grazer Strasse 4, 28359 Bremen, Germany; 2 Health Sciences Bremen, University of Bremen, Bremen, Germany; 3 Cooperative Research Group for Evidence-Based Public Health, Institute of Public Health and Nursing Research, and Leibniz Institute for Prevention Research and Epidemiology-BIPS GmbH, University of Bremen, Achterstr. 30, 28359 Bremen, Germany

**Keywords:** Secondhand smoke exposure, Public smoking ban, Tobacco control, Smoke-free legislation, Displacement, Meta-analysis

## Abstract

**Backround:**

Meta-analysis of the impact of public smoking bans on children’s exposure to secondhand smoke (SHS) exposure at home.

**Methods:**

The electronic databases of PubMed, Web of Science, PsycINFo, ASSIA, CINAHL were searched. German public health journals not captured by these databases and grey literature were considered in addition.

Studies were included when they reported children’s SHS exposure at home in relation to smoke-free legislation by measuring exposure before and after the introduction of a public smoking ban. Studies had to provide results on exposure prevalences of children aged below 18 years. Language of publications was restricted to German and English.

Details of the included studies (*n* = 15) were extracted by one author and checked for accuracy by a second author. Given the exposure prevalences before and after the introduction of a smoke-free legislation, a random-effects meta-analysis of relative risks (RR) was conducted. Results were presented in a forest plot.

**Results:**

Meta-analysis showed that the overall effect was a decreased exposure to SHS in the children’s homes after introduction of a public smoking ban (RR = 0.72; 95% CI = 0.62–0.83). Only two of the 15 studies indicated an increased exposure. Sensitivity analyses considering the type of smoke-free legislation, children’s age group and study quality did not substantially alter the result.

**Conclusion:**

The assumption of a displacement of smoking into homes with children due to smoke-free legislation in public places could not be confirmed. Additional research is needed to analyse long-term trends.

**Electronic supplementary material:**

The online version of this article (10.1186/s12889-018-5679-z) contains supplementary material, which is available to authorized users.

## Background

Secondhand smoke (SHS) is known to consist of several harmful substances relevant for health [1]. Several adverse health effects for non-smokers are associated with SHS, including multiple types of cancer, cardiovascular disease and/or acute respiratory illness [2, 3]. Worldwide, SHS is regarded as the third leading cause of preventable disease, disability and death [2] and approximately 600,000 non-smokers die per year due to SHS exposure [4]. Because especially young children are unable to protect themselves from the risks of SHS exposure, they are the most tragically affected group. An estimated 40–50% of children with smoking parents and/or other smoking household members are regularly exposed to SHS [5].

Since the 1980′ smoke-free legislations and restricted places where people are allowed to smoke increased [6, 7]. and with the WHO Framework Convention on Tobacco Control (FCTC) from 2005 the first global public health treaty came into effect. FCTC article 8 comprises smoke-free legislation in terms of smoking bans at public places aiming to reduce exposure to secondhand smoke among non-smokers [8].

With the introduction of public smoking bans in enclosed spaces of hospitality venues and further workplaces, the so-called displacement hypothesis has been put forward. This proposes that smoke-free laws may result in a displacement from smoking in public to smoking at home [9, 10]. If proven, this would contradict one of the main aims of public smoking bans, as it would have a deleterious effect on health especially for children with smoking parents and/or family members. The negative effects of smoking within the home go beyond the immediate health risks of exposure to SHS. Children living with an adult smoker are up to twice as likely to take up smoking themselves [11, 12].

However, there is a growing body of evidence that supports the rejection of the displacement hypothesis, and instead supports the alternative social diffusion hypothesis which suggests that changes in social norms due to public smoking bans may result in an increase in voluntary home smoking bans [6, 7, 13–15]. Previous studies [6, 7, 16] demonstrated an effect of public smoking bans by showing a decrease in smoking in bars and restaurants after the introduction of public smoking bans, but found no changes in smoking behaviour at home. Edwards et al. [17] even came to the conclusion that there was a significant reduction in SHS exposure in houses or flats.

Apparently previous research indicates a general rejection of the displacement hypothesis, but the focus was not specifically on SHS exposure of children at home before and after the introduction of smoke-free legislation. Therefore, this review aims to determine the overall impact of public smoking bans on children’s SHS exposure at home.

## Methods

This systematic review follows the PRISMA statement for systematic reviews and meta-analyses (Additional file 1) [18]. The study was registered with the PROSPERO international prospective register of systematic reviews (Registration no. CRD42017059522).

### Literature search

The search syntax was applied in June 2016 to the following electronic databases: Web of Science, PsycINFO (via Ovid), PubMed, Applied Social Sciences Index and Abstracts (ASSIA) (via ProQuest), and the Cumulative Index to Nursing and Allied Health Literature (CINAHL) (via EBSCO Host). It was developed using keywords from other systematic reviews that were previously performed. The keywords included in the final search syntax were related to: (1) tobacco smoke, (2) smoking ban, (3) secondhand smoke and (4) children. An example of the search syntax applied to title and abstract is shown in Additional file 2. The search was restricted to German and English language articles only, with no further restrictions on e.g. publication year. An abbreviated version of the search strategy was entered into the database OAlster for grey literature. Furthermore a manual search for additional articles was conducted in the two German public health journals *Prävention – Zeitschrift für Gesundheitsförderung* (Prevention - Journal for Health Promotion [own translation]) and *Prävention und Gesundheitsförderung* (Prevention and Health Promotion [own translation]), which are not captured by the aforementioned databases. The reasons for including German language publications were that smoke-free legislations of varying degrees of comprehensiveness have been implemented in Germany. Smoke-free legislation in Germany is governed at the federal state level resulting in marked differences in extent of protection against tobacco smoke. As international journals were less likely to publish studies in effects of a federal state legislation German language publications were included in addition.

Since 2003, an annual conference on tobacco control has been organized by the WHO collaborating center for tobacco control at the German Cancer Research Center (*Deutsches Krebsforschungszentrum*; DKFZ). Presentations and programs of these conferences were examined for additional international or national relevant studies [19]. Finally, references of all articles fulfilling the eligibility criteria were checked for further relevant studies. EndNote X7.5 was used to organize the included studies.

### Selection process

After removing duplicates, two reviewers independently screened title and abstracts for the eligibility criteria.. When there was clear evidence for irrelevance, studies were excluded. Full texts of all potentially relevant articles were checked for eligibility by one author (SN) and finally included after discussion with another author (GB).

### Eligibility criteria

Studies were defined as eligible when they were based on smoke-free legislations in terms of smoking bans in public places (e.g. hospitality venues, further workplaces). Internal regulations like school policies were not considered. Moreover, there had to be a before-after-design in terms of at least two successive cross-sectional studies or a cohort study to compare the SHS exposure of children at home before and after the smoke-free legislation came into effect. Studies reporting private smoking restrictions in the home and studies with comparisons of countries with and without smoke-free legislation with no before-after data were not included. All types of quantitative studies were included as long as they reported on SHS exposure prevalence for children aged under 18 years. Data could be collected via self-report of exposure (by children themselves or their parents), by analysis of biomarkers, such as nicotine or cotinine, by indoor air pollution measurement or by counting cigarette butts or ash trays. Studies comparing households with and without children were excluded. Finally, only primary articles were included.

### Evaluation of included studies

The data extraction was performed by the first author (SN) and checked for accuracy for all studies by the last author (GB). A pre-defined table was used for the evaluation of the included studies. It contained information about study design, type of SHS exposure, measurement of exposure prevalence and main results. Studies were classified as evaluating comprehensive smoke-free law or mixed smoke-free law, according to the form of legislation that was introduced in the different countries the studies reported on. The category comprehensive smoke-free law stands for a public smoking ban that affects the whole country and offers no opportunity of smoking in any hospitality venue, whereas the category mixed smoke-free law comprises countries with regional differences in the type or extent of public smoking bans within a country or with an exceptional rule for certain types of hospitality venues such as small bars.

A modified version of the quality checklist proposed by Ogilvie et al. [20] was used for quality assessment. The tool consisted of a five-item checklist to assess several risks of bias and methodological quality criteria of each study and a three-level scale to evaluate the suitability of study design (Additional file 3). The quality of the included studies was checked by two authors (SN, GB) independently. A study was rated high quality when at least 4 out of 5 points of the methodological quality criteria were applied. Any disagreements were discussed to achieve consensus and a final result.

### Statistical methods for meta-analysis

All included studies presented proportions of children exposed to SHS at home before and after introduction of smoke-free legislation. When sample sizes of all examined children were reported for both before and after groups, sample sizes of exposed children were calculated using these proportions. Given these values, the relative risk (RR) of being exposed to SHS after the introduction of smoke-free legislation compared to before legislation was calculated. A meta-analysis was conducted to synthesize the results. A pooled RR with 95% confidence interval (CI) was calculated to see how the risk of being exposed to SHS changed after the introduction of a public smoking ban. The I^2^ statistic for heterogeneity was calculated to determine the proportion of between-study variation [21]. A random effects model using DerSimonian-Laird estimator for τ^2^ was used for the pooled RR because the I^2^ test of heterogeneity was highly significant (*p* < 0.001) [22]. Sensitivity analyses were performed to explore possible causes of heterogeneity.

A funnel plot [23] was used to check whether there could have been publication bias. The analysis was done with R version 3.3.1 using the metafor package [24].

## Results

After removing duplicates, 3037 potentially eligible articles were identified in the systematic search. Finally, 15 studies [10, 25–38] were included in this systematic review with regard to the eligibility criteria. If several articles had been published based on the same data of a single study, these articles were considered as one study. This was the case for two studies [29, 39]. The only exceptions to this were articles reporting on different (independent) regions within the same country, having different smoke-free policies. As long as the data were available, these articles were included in the analysis as separate studies. This was the case for two studies reporting on the CHETS survey for Scotland and Wales [32, 34]. Articles reporting on the same data source but with different data collection periods, lengths of follow-up or different age groups, were also included in the analysis as separate studies. This was the case for three studies reporting on the Health Survey for England [26, 30, 33].

**Fig. 1:**
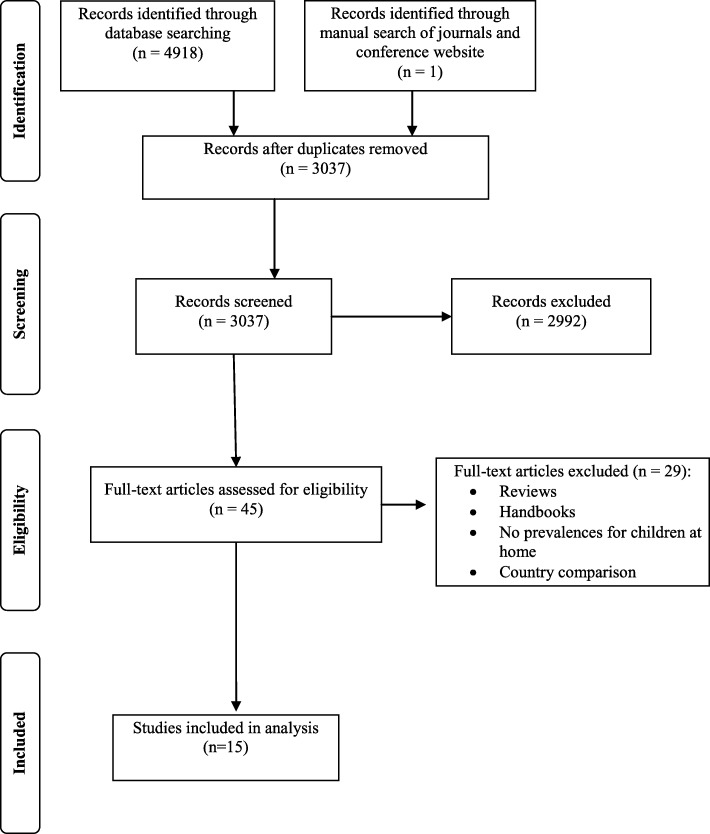
PRISMA Flow diagram of study selection

The entire search strategy can be reconstructed from the PRISMA Flow diagram (Fig. 1).

### Study characteristics

Of the 15 studies, 14 were repeated cross-sectional studies [10, 25–27, 29–38] and one was a prospective cohort study [28]. Five Studies were conducted in Great Britain [26, 30, 32–34]. Only two studies had a follow-up period longer than one year [25, 38]. The sample size was very large in most of the studies, ranging from 118 [28] to 68,000 [35] participants. Most of the children were aged between five and 15 years. Eight studies reported on cotinine levels [10, 26, 28–30, 32–34]. All studies were published between 2007 and 2016. Characteristics of the included studies are summarized in Table 1.

**Table 1:** Characteristics of studies included in the meta-analysis (*n* = 15)

First author	Location	Study design	Data collection	Age of children (years)	Exposure	Measurement of exposure prevalence used for meta-analysis	Main results
Comprehensive Smoke-Free Law							
Kabir et al. (2010) [25]	Ireland Legislation: 2004	Repeated cross-sectional, N = app. 10,640 children	ISAAC (1995, 1998, 2002, 2003, 2007) Follow-up period: 1 measurement after approx. 3 years	13–14	Parental smoking at home. Measurement: Q	Reported prevalence’s for exposure in each survey year	Q: SHS exposure at home remained unchanged.
Sims et al. (2012) [26]	England Legislation: 2007	Repeated cross-sectional, *N* = 23,680 children	HSE (1996, 1997, 1998, 2001, 2002, 2003, 2005, 2006, 2007, 2008) Follow-up period: 1 measurement after approx. 1 year	4–15	Parental smoking at home. Measurement: Q, CL (more than 1.7 ng/ml)	Reported proportions of household smoking status	Q and CL:SHS exposure at home decreased. (CL more than 1.7 ng/ml)
Huang et al. (2012) [27]	Southern Taiwan Legislation: 2009	Cross-sectional, *N* = 4450 children	Control of School-aged Children Smoking Study Survey (2008,2009) Follow-up period: 1 measurement after few months	8–13	Smoking children, family members or visitors at home. Measurement: Q	Reported changes in proportions of household SHS exposure (summed up for men and women and None, 1–3 or 4+ days/ week)	Q: SHS exposure at home remained unchanged.
Fernández et al. (2015) [28]	Spain Legislation: 2011	Prospective cohort study, *N* = 118 boys and their parents	INMA-Network (2005–2006, 2011–2012) Follow-up period: 1 measurement after approx. 1 year	4–5	Parents (mother, father, both) smoking at home. Measurement: Q, CL	Reported proportions of exposure to SHS at home (yes, at least one smoker)	Q and CL: SHS exposure at home increased.
Ho et al. (2010) [10]	Hong Kong Legislation: 2007	Cross-sectional, N_06 = 3243 N_08 = 4965 children	Data from randomly selected schools (2006, 2008) Follow-up period: 1 measurement after approx. 1 year	7–10	Parental smoking at home. Measurement: Q, HNC	Reported prevalences of SHS exposure based on HNC (summarized: None, 1–3, 4–7, any days exposed)	Q with HNC: SHS exposure at home increased.
Chan et al. (2014) [29]	Hong Kong Legislation: 2007	Cross-sectional, (RCT), N_(05–06) = 333 N (07–08) = 742	Data from the maternal/child/Student Health Centers (2005–2006, 2007–2008) Follow-up period: 1 measurement after approx. 1 year	0–6	Smoking father at home. Measurement: Q, HNC	Reported proportions of children’s exposure by smoking father reported by mother	Q and HNC: SHS exposure at home decreased.
Jarvis et al. (2012) [30]	England Legislation: 2007	Repeated cross-sectional, *N* = 10,825 children	HSE (1998, 2001, 2002, 2003, 2005, 2006, 2007, 2008) Follow-up period: 1 measurement after approx. 1 year	4–15	Smoking parents or any other people at home. Measurement: I, CL	Reported proportions of children with smoking parents.	I and CL: SHS exposure at home decreased slightly.
Bolte et al. (2015) [31]	Germany (Bavaria), Legislation: 2008	Repeated cross-sectional, *N* = 17,892 parents with their children	Health monitoring units (GME) (2004–2005, 2005–2006, 2008–2009), Follow-up period: 1 measurement after approx. 1 year	5–6	Smoking parents at home, in cars. Measurement: I	Reported prevalences of SHS exposure at home	I: SHS exposure at home decreased slightly.
Holliday et al. (2009) [32]	Wales, Legislation: 2007	Repeated cross-sectional, *N* = 3216 children	CHETS (2007,2008) Follow-up period: 1 measurement after approx. 1 year	10–11	Parents or any other people smoking at home. Measurement: Q, CL	Reported prevalences of SHS exposure at home	Q and CL: SHS exposure at home remained unchanged.
Jarvis et al. (2015) [33]	England Legislation: 2007	Repeated cross-sectional, *N* = 13,327 children	HSE (1998, 1999, 2000, 2001, 2002, 2003, 2004, 2005, 2006, 2007, 2008, 2009, 2010, 2011,2012) Follow-up period: 5 measurements during 5 years	0–15	Parents or any other people smoking at home. Measurement: I, CL	Reported percentages of children with smoking parents.	I and CL: SHS exposure at home decreased.
Akhtar et al. (2007) [34]	Scotland Legislation: 2006	Repeated cross-sectional, *N* = 4676 children	CHETS (2006,2007) Follow-up period: 1 measurement after approx. 1 year	10–11	Parents or any other people smoking at home. Measurement: Q, CL	Reported prevalence of SHS exposure at home	Q: SHS exposure at home remained unchanged. CL: Proportion of pupils with higher cotinine remained unchanged, mean cotinine concentration fell by 39%
Mixed Smoke-Free Law							
Sinha et al. (2008) [35]	India Legislation: 2005	Cross-sectional, N_1 = 68,077 N_2 = 12,086 children	GYTS (2003,2006) Follow-up period: 1 measurement after approx. 1 year	13–15	Smoking parents or other smoking people at home. Measurement: Q	Reported exposure prevalences in the week prior to the survey, for each survey year	Q: SHS exposure at home decreased after legislation.
Hawkins et al. (2012) [36]	USA Legislation: NA	Repeated cross-sectional N_1 = 67,607, N_2 = 62,768 families	NSCH (2003,2007) Follow-up period: 1 measurement after approx. 1 year	6–17	Smoking parents at home. Measurement: Q	Reported prevalences of household tobacco use (yes/no) for each survey (only in the text)	Q: SHS exposure at home decreased slightly.
Yao et al. (2016) [37]	USA Legislation: NA	Repeated cross-sectional N_1 = 18,731 children, 44,049 nonsmoking adults	National Health Survey Cancer Control Supplements (2000,2010) Follow-up period: 1 measurement after approx. 1 year	0–17	Smoking parents or other smoking people at home. Measurement: Q	Reported prevalence of SHS exposure at home	Q: SHS exposure at home decreased.
Kuntz et al. (2016) [38]	Germany Legislation: 2008	Cross-sectional, N_1 = 6680 N_2 = 4455 children	KiGGS (2003–2006, 2009–2012) Follow-up period: 1 measurement after approx. 1 year	0–6	Smoking parents or other smoking people at home. Measurement: Q	Reported prevalence of domestic exposure	Q: SHS exposure at home decreased.

*Abbreviations: ISAAC* International study of asthma and allergies in childhood, *CHETS* Child exposure to environmental tobacco smoke, *INMA* Environmental and childhood research network, *HSE* Health survey for England, *GYTS* Global youth tobacco survey, *NSCH* National Survey of children’ health, *KiGGS* German health interview and examination survey for children and adolescents, *Q* Questionnaire, *I* Interview, *CL* Cotinine level (urine, saliva), *HNC* Hair nicotine concentration, *NA* Not applicable

### Quality of the included studies

Detailed results of the quality assessment are presented in Additional file 4. Almost every study only reached the lowest category C for suitability of study design, due to the lack of comparison groups. In contrast, the methodological quality criteria were well covered with the exception of the data collection instruments criterion which was acceptable in only six studies [26, 28, 30, 32–34]. This resulted from the fact that data collection instruments were only seen as reasonable when the studies used biomarkers to assess children’s SHS exposure at home.

### Meta-analysis

**Fig. 2:**
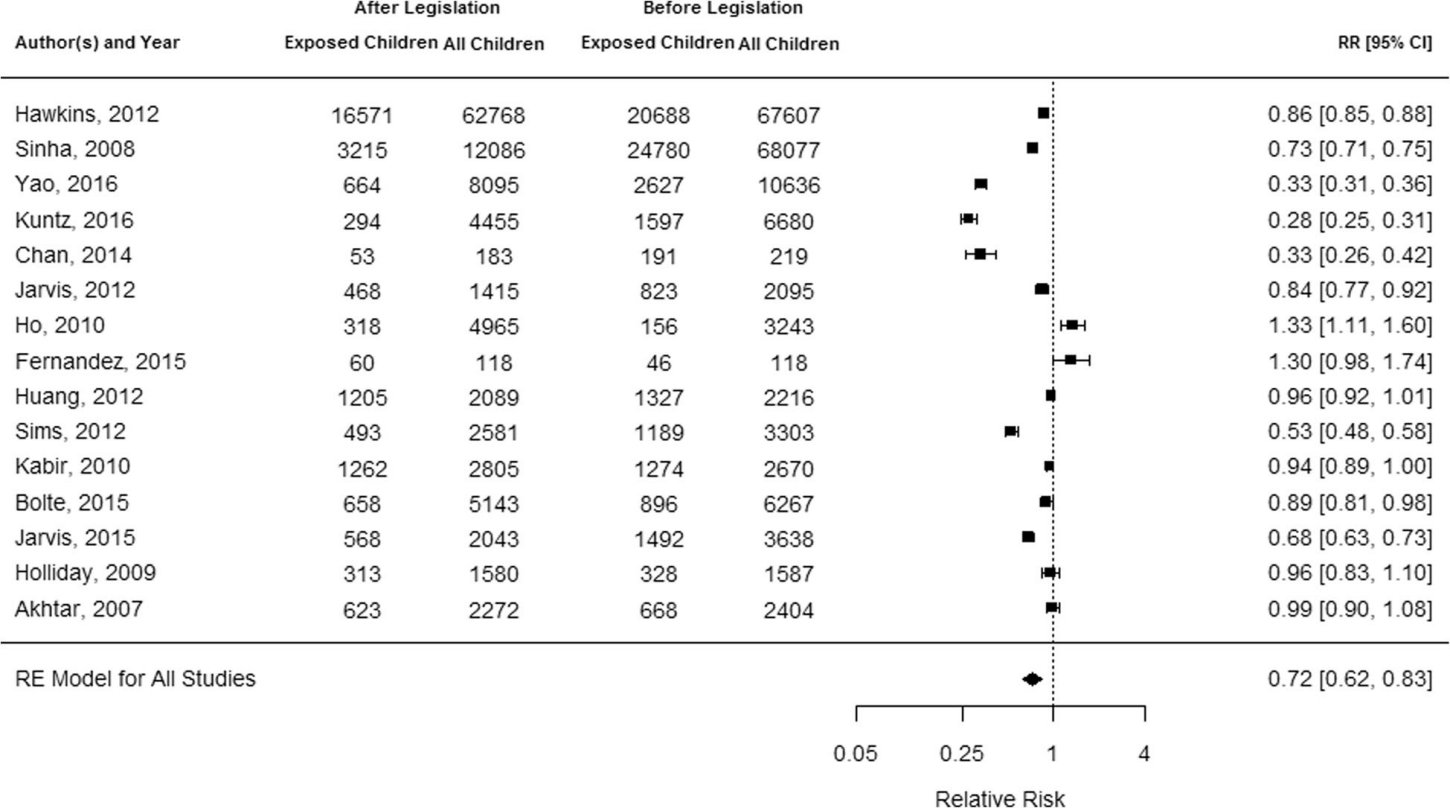
Forest plot summarizing the estimated relative risks of the included studies

The meta-analysis was summarized in a forest plot (Fig. 2). Of the calculated RRs, 14 were based on exposure measurements from parental report [25–38], and one was based on exposure measurement verified by hair nicotine concentration (HNC) [10]. Overall, children had a lower risk of being exposed to SHS at home after the introduction of a public smoking ban (RR = 0.72, 95% CI = 0.62–0.83, *p* < 0.0001). Only 2 studies had a RR above 1 [10, 28] indicating an increased RR after the smoke-free legislation, but only one of these two studies presented significant results [10]. The level of heterogeneity was very high with I^2^ = 98.82%.

Almost all studies gave the same results by measuring SHS exposure before and after smoke-free legislation via self-report or biomarker. Only one study [34] stated that self-reported SHS exposure at home remained unchanged, but the mean cotinine concentration fell by 39%. However, the proportion of children with higher cotinine levels remained unchanged as well.

### Sensitivity analyses

To investigate the sources of heterogeneity and to test the robustness of the results of the meta-analysis, several sensitivity analyses were performed (Additional files 5, 6 and 7). The first sensitivity analysis included only those nine studies with high quality [10, 26, 27, 30, 32–34, 36, 38]. The level of heterogeneity was high (I^2^ = 98.5%) and the effect size was very similar to the overall model (RR = 0.76, 95% CI = 0.63–0.91, *p* = 0.0037). The second sensitivity analysis considered studies with children in special age groups. The first group consisted of three studies [25, 32, 34] including children aged 10–15 years. One study with children with an age range of 4–15 years was excluded [26]. Heterogeneity disappeared completely (I^2^ = 0, *p* = 0.7099) and the effect size decreased (RR = 0.96, 95% CI = 0.91–1.00, *p* = 0.0497) in comparison to the overall model. The second group consisted out of four studies considering children aged 0–6 years [28, 29, 31, 38]. In this case the level of heterogeneity was almost 100% (I^2^ = 99.0%) and the effect size was not significant (RR = 0.57, 95% CI = 0.27–1.22, *p* = 0.1439). The third sensitivity analysis compared studies with comprehensive smoke-free law and with mixed smoke-free law. 11 studies with comprehensive law were included [10, 25–34]. Heterogeneity was high (I^2^ = 96.4%) whereas the effect size decreased slightly (RR = 0.83, 95%CI = 0.72–0.96, *p* = 0.0155, *p* < 0.001). In contrast, the four studies [35–38] with mixed smoke-free laws produced a more pronounced effect size compared to the overall model (RR = 0.49, CI = 0.35–0.69); however, heterogeneity was high (I^2^ = 99.7%).

As mentioned before, three studies [26, 30, 33] reported on results of the Health Survey of England and overlapped in baseline and follow-up periods. Therefore, an additional meta-analysis including only the one study with the highest quality [33] was conducted. However, the overall result (RR = 0.73, 95% CI = 0.62–0.85, *p* < 0.0001) did not greatly differ from the above-mentioned effect size.

## Discussion

This meta-analysis shows that children’s SHS exposure at home did not increase after the introduction of public smoking bans. Therefore, the displacement hypothesis was not confirmed. Of the 15 studies, only 2 reported increased values for SHS exposure at home, however, 1 of these 2 studies had only 118 participants [10, 28]. Nevertheless, the results must be interpreted with caution due to high heterogeneity between the studies. Sensitivity analyses showed that heterogeneity still remained in the different age groups, in mixed or comprehensive law groups and in quality-specific groups. The comprehensive and mixed smoke-free law yielded reductions of SHS exposure at home, whereby the effect was much greater in the group with mixed smoke-free laws. However, the four studies [35–38] in this group had a wide range of RRs (Fig. 2). As mixed smoke-free laws included laws with exemptions and laws that varied across regions, there might also be a difference of the impact on SHS exposure within this category. The number of four studies was too small to further explore potential differences, more studies are needed to prove an actual effect of these different ways of implementation of tobacco control measures.

Another possible cause of heterogeneity in the findings between the studies were differences in the way how children’s SHS exposure at home was assessed. The main reasons for heterogeneity are the various ways to assess SHS exposure and to report on exposure proportions. First, each study used a different framework to define the household smoking status. Some studies only distinguished between “Yes, someone smokes regularly” and “No, no-one smokes” [26]. Others built two or more categories, such as 1–3, 4–7 or any days exposed [27] or 1, 2–4, 5–7, and 8–10 h a day [29]. Second, not all included studies considered the smoking behaviour of all household members in their analysis. In some studies, only the parents were considered for SHS exposure at home (e.g. Kabir et al. [25], Fernandez et al. [28]), whereas other studies also included smoking visitors (e.g. Jarvis et al. [33], Holliday et al. [32]). One study [29] only used mother-reported values of smoking fathers. In addition, smoking within a family’s home was defined differently concerning the relevant parts of a flat. One study [31] included smoking on a balcony independently from smoking inside the flat into the definition of SHS exposure, because children are exposed to all SHS ingredients which transiently adhere to clothes [40]. Other studies only considered smoking inside the flat or house.

Besides sensitivity analysis, meta-regression might have been another reasonable way of exploring heterogeneity. This meta-analysis rests upon published data of statistical analyses. Due to the lack of data from the primary studies such as the estimated treatment effect, its variance, and covariate values for each study a meta-regression was not possible.

The funnel plot (Additional file 8) shows that most of the studies had a substantial sample size. The effects of two studies with the smallest sample size [28, 29] are widespread around the overall effect of the analyses, which is an indicator against publication bias. Nevertheless, the studies on the top of the funnel plot are mostly located asymmetrically on the right side of the overall effect [41]. The search strategy of the present review contained grey literature in order to reduce potential publication bias when considering only publications in peer-reviewed journals.

### Other research

To our knowledge, this is the first systematic review to focus on children’s SHS exposure at home before and after the introduction of smoke-free legislations, based on conducting a meta-analysis of pooled relative risks.

Borland et al. [42] searched for determinants of smoke-free homes in Canada, the UK, USA and Australia within the International Tobacco Control (ITC) Four Country Survey (ITC-4). Main predictors for a reported smoke-free home or introduced home smoking bans were having a child, especially a young child, and having another non-smoking adult in the household. Mons et al. used similar data from the International Tobacco Control Policy Evaluation Project Europe surveys and tried to find changes in predictors or changes of home smoking bans after the introduction of smoke-free laws in Ireland, France, Germany and the Netherlands. The authors confirm having young children as a main predictor of having a pre-legislation home smoking ban. In addition, people who supported smoking bans in bars were more likely to introduce home smoking bans [43]. These results support the findings of the present review which came to the result that more than half of the studies reported a reduction of children’s SHS exposure at home. A study from New Zealand also evaluated the impact of a national smoke-free law and concluded that the reported SHS exposure at home was significantly reduced [17]. Callinan et al. conducted a review with 50 included studies and found no change of reported SHS exposure at home [6]. However, neither of these latter studies focused on children [6, 17].

### Strengths and limitations

The present review as well as the included studies have some limitations that need to be addressed. Only a small number of 15 studies were included in the meta-analysis. Especially the category of studies presenting mixed smoke-free laws comprised only four studies [35–38]. In addition to the small number of studies, 5 of the 15 studies were conducted in Great Britain [26, 30, 32–34]. It would be interesting to have a greater variation of included countries to see if the results of the meta-analysis remained stable.

14 of the 15 included studies were cross-sectional studies. Measuring exposure prevalences before and after the introduction of public smoking bans using a cohort study design would be preferable as varying samples of children included in a repeated cross-sectional design might bias the results.

For most of the included studies the suitability of study design was the lowest category (i.e. C), with exception of the study of Jarvis et al. [33] who reached category B. Nevertheless, nine studies fulfilled four or more out of five of the methodological quality criteria [10, 26, 27, 30, 32–34, 36, 38].

Another limitation might be the use of self-reports for collecting data on children’s SHS exposure at home. In 14 out of 15 studies children’s exposure to SHS was measured based on parents self-reports. This measure may be particularly prone to social desirability bias and hence exaggerate the reduction in children’s SHS exposure - particularly if the diffusion hypothesis is correct and smoke-free legislation results in changed social norms about smoking in the present of children [15]. Therefore, as methodological quality criterion of credibility of data collection instruments within the applied quality checklist, any biochemical measurement of SHS exposure (such as the validated specific and sensitive biomarker cotinine [44]) was assumed to be of higher quality than parental reports. Nevertheless, cotinine gives information about total SHS exposure which may not only occur within the child’s home. In addition, parental reports about SHS exposure answered by parents are essentially valid [45, 46]. Thus, it is assumed that data collection by parental report did not substantially bias the results on children’s SHS exposure at home. Our assumption is in line with the observation that the impact of the smoke-free legislation on children’s SHS exposure was irrespective of method of exposure assessment (self-report vs. biomarker).

The short period of follow-up after the introduction of smoke-free legislation is another limitation. Repeated measurements over a longer follow-up period might reveal more information about when and how tobacco control policies influenced the parental smoking behaviour in a child’s home [15]. Long-term effects could not be considered because only two studies [25, 33] had a follow-up period longer than one year after the introduction of a smoke-free legislation. Nevertheless, even these two studies concluded that there is no impact at all [25] or a positive impact [33] of smoke-free legislation on children’s SHS exposure at home. Just recently, a further follow-up period of four years on the effects of the comprehensive smoking ban in Bavaria, Germany, on children’s SHS exposure at home first examined by Bolte et al. has been published [47]. This further follow-up demonstrated a positive impact on children’s SHS exposure at home.

Only two studies [35, 36] took other tobacco control policies such as increasing prices and advertising into account in their analysis which has been introduced at the same time as the smoke-free legislation. The effects of public smoking bans might be moderated by other policies actually contributing to changes of SHS exposure at home. Moreover, not only the combination of several tobacco control measures but even the societal debate might have had an impact. For example, Jarvis et al. [33] stated that the actual starting point of decreasing SHS exposure in England was not the introduction of a smoke-free legislation, but the intense national debate on the implementation of a smoke-free law. For the studies included in the present meta-analysis, it would be interesting to know whether the cross-sectional studies performed before smoke-free legislation came into force took place during times of societal disputes about tobacco control and the protection of non-smokers.

The present review also has some strengths. First, the meta-analysis had considerable sensitivity. By converting proportions into relative risks, every effect from each study can be compared on the same level. The broad range of literature search is also a strength of this review. Including a manual search in journals not covered by the electronic databases and also the grey literature, helped to identify as many eligible studies as possible. A strength of most of the included studies was the large sample size.

## Conclusion

The present meta-analysis shows that up to now there is no indication of displacement of smoking into homes after the introduction of smoke-free legislations. However, since only 15 studies were included in the analyses, additional studies are required to assess the impact of public smoking bans on children’s SHS exposure at home. More detailed results might be achieved if other tobacco control measures and their effects on SHS exposure at home are also taken into account. Finally, as most of the studies had a short follow-up period, long-term studies on this topic are also required.

## Additional files

Additional file 1: PRISMA-Equity 2012 Extension: Reporting Guidelines for Systematic Reviews with a Focus on Health Equity. This file contains the responses to the PRISMA-Equity 2012 Extension check-list. (DOCX 20 kb)

Additional file 2: Sample search string for PubMed MEDLINE. This file contains a sample search string for PubMed MEDLINE. (DOCX 13 kb)

Additional file 3: Suitability of study design and methodological quality criteria. This file contains a check-list to evaluate the suitability of study design and the methodological quality criteria of the studies included in the meta-analysis. (DOCX 19 kb)

Additional file 4: Results from the quality assessment. This file contains a table presenting the results of the quality assessment of the studies included in the meta-analysis. (DOCX 17 kb)

Additional file 5: Sensitivity analysis for studies with good quality. Forest plot summarizing the estimated relative risks of the included studies. (TIF 522 kb)

Additional file 6: Sensitivity analysis for different age groups. Forest plot summarizing the estimated relative risks of the included studies. (TIF 522 kb)

Additional file 7: Sensitivity analysis for studies with different smoke-free policies. Forest plot summarizing the estimated relative risks of the included studies. (TIF 522 kb)

Additional file 8: Funnel plot. This file contains the funnel plot resulting from the included studies in the meta-analysis. It is an instrument for detecting publication bias. (TIF 611 kb)

## Abbrevations

ASSIA: Applied Social Science Index and Abstracts
CHETS: Child Exposure to Environmental Tobacco Smoke
CI: Confidence interval
CINAHL: Cumulative Index to Nursing and Allied Health Literature
DKFZ: Deutsches Krebsforschungszentrum
FCTC: Framework Convention on Tobacco Control
HNC: Hair cotinine concentration
ITC: International Tobacco Control
ITC-4: International Tobacco Control Four Country Survey
RR: Relative risk
SHS: Secondhand smoke
WHO: World Health Organization
