# Supplementary material for: Impact of public smoking bans on children’s exposure to tobacco smoke at home: a systematic review and meta-analysis
Source: BMC Public Health. 2018 Jun 21;18:749. doi: 10.1186/s12889-018-5679-z (PMC6011268; doi:10.1186/s12889-018-5679-z)
Supplement: Supplementary file 1 — PRISMA-Equity 2012 Extension: Reporting Guidelines for Systematic Reviews with a Focus on Health Equity. This file contains the responses to the PRISMA-Equity 2012 Extension check-list. (DOCX 20 kb) [file 12889_2018_5679_MOESM1_ESM.docx]

**Additional file 1: PRISMA-Statement: Preferred Reporting Items for Systematic Reviews and Meta-Analyses.**Responses are written in bold types and displayed in brackets.

| **Section** | **Item** | **Standard PRISMA Item** |
| --- | --- | --- |
| TITLE |  |  |
| Title | 1 | Identify the report as a systematic review, meta-analysis, or both.  **(Title)** |
| ABSTRACT |  |  |
| Structured summary | 2 | Provide a structured summary including, as applicable: background; objectives; data sources; study eligibility criteria, participants, and interventions; study appraisal and synthesis methods; results; limitations; conclusions and implications of key findings; systematic review registration number. **(Abstract)** |
| INTRODUCTION |  |  |
| Rationale | 3 | Describe the rationale for the review in the context of what is already known. **(Introduction)** |
| Objectives | 4 | Provide an explicit statement of questions being addressed with reference to participants, interventions, comparisons, outcomes, and study design (PICOS). **(Introduction)** |
| METHODS |  |  |
| Protocol and registration | 5 | Indicate if a review protocol exists, if and where it can be accessed (e.g., Web address), and, if available, provide registration information including registration number. **(Methods)** |
| Eligibility criteria | 6 | Specify study characteristics (e.g., PICOS, length of follow-up) and report characteristics (e.g., years considered, language, publication status) used as criteria for eligibility, giving rationale.  **(Methods: Eligibility criteria)** |
| Information sources | 7 | Describe all information sources (e.g., databases with dates of coverage, contact with study authors to identify additional studies) in the search and date last searched.  **(Methods: Literature search)** |
| Search | 8 | Present full electronic search strategy for at least one database, including any limits used, such that it could be repeated.  **(Supplementary file 2)** |
| Study selection | 9 | State the process for selecting studies (i.e., screening, eligibility, included in systematic review, and, if applicable, included in the meta-analysis).  **(Methods: Selection process, Eligibility criteria, Evaluation of included studies, Synthesis of results)** |
| Data collection process | 10 | Describe method of data extraction from reports (e.g., piloted forms, independently, in duplicate) and any processes for obtaining and confirming data from investigators.  **(Methods: Evaluation of included studies)** |
| Data items | 11 | List and define all variables for which data were sought (e.g., PICOS, funding sources) and any assumptions and simplifications made. **(Methods: Evaluation of included studies)** |
| Risk of bias in individual studies | 12 | Describe methods used for assessing risk of bias of individual studies (including specification of whether this was done at the study or outcome level), and how this information is to be used in any data synthesis.  **(Methods: Evaluation of included studies, supplementary file 3)** |
| Summary measures | 13 | State the principal summary measures (e.g., risk ratio, difference in means). **(Methods: Statistical methods for the meta-analysis)** |
| Synthesis of results | 14 | Describe the methods of handling data and combining results of studies, if done, including measures of consistency (e.g., I^2^) for each meta-analysis. **(Methods: Statistical methods for the meta-analysis)** |
| Risk of bias across studies | 15 | Specify any assessment of risk of bias that may affect the cumulative evidence (e.g., publication bias, selective reporting within studies). **(Methods: Statistical methods for the meta-analysis)** |
| Additional analyses | 16 | Describe methods of additional analyses (e.g., sensitivity or subgroup analyses, meta-regression), if done, indicating which were pre-specified. **(Methods: Statistical methods for the meta-analysis)** |
| RESULTS |  |  |
| Study selection | 17 | Give numbers of studies screened, assessed for eligibility, and included in the review, with reasons for exclusions at each stage, ideally with a flow diagram. **(Results, Figure 1)** |
| Study characteristics | 18 | For each study, present characteristics for which data were extracted (e.g., study size, PICOS, follow-up period) and provide the citations. **(Table 1)** |
| Risk of bias within studies | 19 | Present data on risk of bias of each study and, if available, any outcome level assessment (see item 12).  **(Supplementary File 4)** |
| Results of individual studies | 20 | For all outcomes considered (benefits or harms), present, for each study: (a) simple summary data for each intervention group (b) effect estimates and confidence intervals, ideally with a forest plot. **(Table 1, figure 2)** |
| Synthesis of results | 21 | Present results of each meta-analysis done, including confidence intervals and measures of consistency. **(Results: Meta-analysis, figure 2)** |
| Risk of bias across studies | 22 | Present results of any assessment of risk of bias across studies (see Item 15). **(Supplementary file 8)** |
| Additional analysis | 23 | Give results of additional analyses, if done (e.g., sensitivity or subgroup analyses, meta-regression [see Item 16]).  **(Results: Sensitivity analysis)** |
| DISCUSSION |  |  |
| Summary of evidence | 24 | Summarize the main findings including the strength of evidence for each main outcome; consider their relevance to key groups (e.g., healthcare providers, users, and policy makers).  **(Discussion)** |
| Limitations | 25 | Discuss limitations at study and outcome level (e.g., risk of bias), and at review-level (e.g., incomplete retrieval of identified research, reporting bias). **(Discussion: Strengths and limitations of this review, Strengths and limitations of the closer selected studies)** |
| Conclusions | 26 | Provide a general interpretation of the results in the context of other evidence, and implications for future research.  **(Discussion:** **Other research, Future look/Conclusions)** |
| FUNDING |  |  |
| Funding | 27 | Describe sources of funding for the systematic review and other support (e.g., supply of data); role of funders for the systematic review. **(Declarations: Funding)** |

**Abbreviations:** NA = Not Applicable.

Adopted and modified from: Welch V, Petticrew M, Tugwell P, Moher D, O'Neill J, Waters E, et al. PRISMA-Equity 2012 extension: reporting guidelines for systematic reviews with a focus on health equity. PLoS Med. 2012;9(10):e1001333.
