# Supplementary material for: Impact of public smoking bans on children’s exposure to tobacco smoke at home: a systematic review and meta-analysis
Source: BMC Public Health. 2018 Jun 21;18:749. doi: 10.1186/s12889-018-5679-z (PMC6011268; doi:10.1186/s12889-018-5679-z)
Supplement: Supplementary file 2 — Sample search string for PubMed MEDLINE. This file contains a sample search string for PubMed MEDLINE. (DOCX 13 kb) [file 12889_2018_5679_MOESM2_ESM.docx]

**Additional File 2: Sample search string for PubMed MEDLINE**

This file contains a sample search string for PubMed MEDLINE.

Truncation symbol: * = all possible word endings included

(tobacco[Title/Abstract] OR smok*[Title/Abstract] OR anti smok*[Title/Abstract] OR nicotine[Title/Abstract] OR cigar*[Title/Abstract]) AND (ban[Title/Abstract] OR bans[Title/Abstract] OR law*[Title/Abstract] OR legislat*[Title/Abstract] OR polic*[Title/Abstract] OR restrict*[Title/Abstract] OR prohibit*[Title/Abstract]) AND (secondhand[Title/Abstract] OR second hand[Title/Abstract] OR secondhand smoke[Title/Abstract] OR shs[Title/Abstract] OR environmental tobacco smoke[Title/Abstract] OR ets[Title/Abstract] OR passive smok*[Title/Abstract] OR involuntary smoking[Title/Abstract] OR cotinine[Title/Abstract] OR exposure[Title/Abstract] OR exposed[Title/Abstract]) AND (child*[Title/Abstract] OR youth*[Title/Abstract] OR adolescent*[Title/Abstract] OR infant*[Title/Abstract] OR newborn*[Title/Abstract] OR juvenile*[Title/Abstract] OR kid*[Title/Abstract] OR offspring[Title/Abstract]) AND ( ( German[lang] OR English[lang] ) )
