# Supplementary material for: Impact of public smoking bans on children’s exposure to tobacco smoke at home: a systematic review and meta-analysis
Source: BMC Public Health. 2018 Jun 21;18:749. doi: 10.1186/s12889-018-5679-z (PMC6011268; doi:10.1186/s12889-018-5679-z)
Supplement: Supplementary file 3 — Suitability of study design and methodological quality criteria. This file contains a check-list to evaluate the suitability of study design and the methodological quality criteria of the studies included in the meta-analysis. (DOCX 19 kb) [file 12889_2018_5679_MOESM3_ESM.docx]

**Additional file 3**

**Suitability of study design and methodological quality criteria**

This file contains a check-list to evaluate the suitability of study design and the methodological quality criteria of the included studies.

| **Description** | |
| --- | --- |
| **Suitability of study design** | |
| Category A | The study design included concurrent comparison groups AND prospective measurement of exposure and outcome. |
| Category B | The study design includes at least two ‘before measurements and at least two ‘after measurements’ but no concurrent comparison group. |
| Category C | The study design involves single ‘before’ and ‘after’ measurements with no concurrent comparison group. |
| **Methodological quality criteria** | |
| Representativeness | Were the study samples randomly recruited from the study population with a response rate of at least 60% OR were they otherwise shown to be representative of the study population? |
| Comparability | Were the baseline characteristics of the comparison groups comparable OR if there were important differences in potential confounders were these appropriately adjusted for in the analysis? For studies without comparison group: Were important confounding factors appropriately controlled for in the analyses? |
| Credibility of data collection instruments | Were data collection tools shown to be credible, e.g. shown to be valid and reliable in published research, OR in a pilot study, OR taken from a published national survey, OR recognized as an acceptable measure (such as biochemical measures of smoking). |
| Attrition rate | Were outcomes studied in a panel of respondents with an attrition rate of less than 30% OR were results based on a cross-sectional design with at least 200 participants included in analysis in each wave? |
| Attributability to intervention | Is it reasonably likely that the observed effects were attributable to the intervention under investigation? This criterion cannot be met if there is evidence of contamination of a control group in a controlled study. Equally, in all types of study, if there is evidence of a concurrent intervention that could also have explained the observed effects and was not adjusted for in analysis, this criterion cannot be met. |

* In the original version proposed by Ogilvie et al., the quality criterion “Comparability” can only be met by studies including a comparison group. For the present review, the criterion was modified so that it could also be met by uncontrolled studies appropriately controlling for possible confounding factors (i.e., by adjusting effect analyses). The criterion “Randomization” was excluded, because RCTs were not possible for the research question. Categories were modified, because category D proposed by Ogilvie et al. did not arise.

**References**Ogilvie D, Fayter D, Petticrew M, Sowden A, Thomas S, Whitehead M et al. The harvest plot: a method for synthesizing evidence about the differential effects of interventions. BMC medical research methodology. 2008;8:8.
