# Supplementary material for: Impact of public smoking bans on children’s exposure to tobacco smoke at home: a systematic review and meta-analysis
Source: BMC Public Health. 2018 Jun 21;18:749. doi: 10.1186/s12889-018-5679-z (PMC6011268; doi:10.1186/s12889-018-5679-z)
Supplement: Supplementary file 4 — Results from the quality assessment. This file contains a table presenting the results of the quality assessment of the studies included in the meta-analysis. (DOCX 17 kb) [file 12889_2018_5679_MOESM4_ESM.docx]

**Additional file 4: Results from the quality assessment**

This file contains a table presenting the results of the quality assessment of the studies included in the meta-analysis.

| **Study** | **Suitability** | | | **Fulfilled criteria** | | | | |
| --- | --- | --- | --- | --- | --- | --- | --- | --- |
|  | A | B | C | Representativeness | Comparability | Credibility of data collection instruments | Attrition rate | Attributability to intervention |
| Akhtar et al. (2007)^34^ |  |  | X | X | X | X | X | X |
| Holliday et al (2009)^32^ |  |  | X | X | X | X | X | X |
| Jarvis et al. (2015)^33^ |  | X |  | X |  | X | X | X |
| Bolte et al. (2015)^31^ |  |  | X | X |  |  | X | X |
| Kabir et al. (2010)^25^ |  |  | X |  |  |  | X | X |
| Sims et al. (2012)^26^ |  |  | X |  | X | X | X | X |
| Huang et al. (2012)^27^ |  |  | X | X | X |  | X | X |
| Fernandéz et al. (2015)^28^ |  |  | X |  | X | X |  | X |
| Ho et al. (2010)^10^ |  |  | X | X | X |  | X | X |
| Jarvis et al. (2012)^30^ |  |  | X | X |  | X | X | X |
| Chan et al. (2014)^29^ |  |  | X |  |  |  |  | X |
| Kuntz et al. (2016)^38^ |  |  | X | X | X |  | X | X |
| Yao et al. (2016)^37^ |  |  | X |  | X |  | X | X |
| Sinha et al. (2008)^35^ |  |  | X | X |  |  | X | X |
| Hawkins et al. (2012)^36^ |  |  | X | X | X |  | X | X |
